# Low-Dose Naltrexone for Excoriation Disorder

**DOI:** 10.7759/cureus.55336

**Published:** 2024-03-01

**Authors:** Kevin Varghese, Xiaofeng Yan, Fei Cao

**Affiliations:** 1 School of Medicine, University of Missouri Kansas City, Kansas City, USA; 2 Psychiatry, University of Missouri Kansas City, Kansas City, USA; 3 Pain Management, University Health Truman Medical Center, Kansas City, USA

**Keywords:** pain management, dermatology, naltrexone, skin-picking, excoriation disorder

## Abstract

Excoriation (skin-picking) disorder (ED) is a condition characterized by the repeated compulsion to pick at the skin, causing physical trauma and psychiatric distress. Patients often desire to cease skin-picking behavior but are unable to do so. Multiple treatment modalities are effective for ED, including naltrexone. Previous reports of naltrexone for ED were at a high dose of 50 mg. The efficacy of low-dose naltrexone (LDN) at 4.5 mg in managing ED has not been reported. We present a case of a 51-year-old female with ED who was evaluated in the pain clinic for fibromyalgia management. Her medications included gabapentin 600 mg PO TID and a history of opioid prescription for diffuse pain. She was started on naltrexone 4.5 mg PO QD for the management of fibromyalgia. Three months later, the patient reported improvement in her skin-picking disorder, with a lessened compulsion to itch at her skin and improved healing of existing lesions. When the naltrexone was temporarily interrupted for an elective procedure, her lesions worsened. Her lesions improved after she resumed the medication. Thereby, this patient experienced a therapeutic benefit from naltrexone for her skin-picking disorder, as demonstrated by the temporal changes in her symptoms. To our knowledge, this is the first reported case of ED improving with LDN, as other cases utilized 50 mg. Though few clinical trials or systematic reviews recommend the use of naltrexone for EDs, our case supports trialing LDN in the appropriate context.

## Introduction

Excoriation (skin-picking) disorder (ED) is a psychogenic condition characterized by recurrent picking at the skin, which leads to lesions and significant functional impairment [1]. Patients are often aware of their behavior but are unable to stop it [1]. Thereby, affected patients may feel shame and lack control [2]. Skin-picking occurs at multiple body sites, including the face, hands, arms, and legs [2]. Onset is often peri-pubertal and more common in females [3]. ED is included in the Diagnostic and Statistical Manual of the American Psychiatric Association fifth edition as an obsessive-compulsive disorder-related disorder [3]. Established treatments include selective serotonin reuptake inhibitors, N-acetyl-cysteine, and adjuvant cognitive behavioral therapy [3]. These treatment modalities are supported by systematic reviews and trials [2,3]. Naltrexone is another treatment option for ED. High-dose naltrexone (HDN) has been demonstrated to be effective for the treatment of ED in previous case reports. Low-dose naltrexone (LDN) for the treatment of ED has not been studied before. Here, we provide a case of a 51-year-old female with ED who benefited from LDN.

## Case presentation

The patient is a 51-year-old female with a past history of depression, fibromyalgia, and excoriation (skin-picking) disorder. She presented to the pain clinic for fibromyalgia management. Her past medications included gabapentin 600 mg PO TID, cyclobenzaprine 5 mg PO TID PRN, and previous opioid prescriptions. Notably, the patient stated that she developed a repetitive skin-picking disorder after being in an abusive relationship. She had never been treated for this ED and took no other psychiatric medications. 

During evaluation, she complained of diffuse pain associated with numbness and tingling in her bilateral hands and feet. The patient described the pain as affecting her quality of life and daily functioning. On examination, she presented with erythematous papules with overlying excoriations and adjacent scarring on the anterior chest, upper back, bilateral arms, and bilateral legs. The patient denied experiencing pruritus. She was started on naltrexone 4.5 mg PO QD to reboot her endogenous opioid system and inhibit neuroinflammation. She was counseled to schedule a psychiatry appointment and see pain management in three months. 

**Figure 1:**
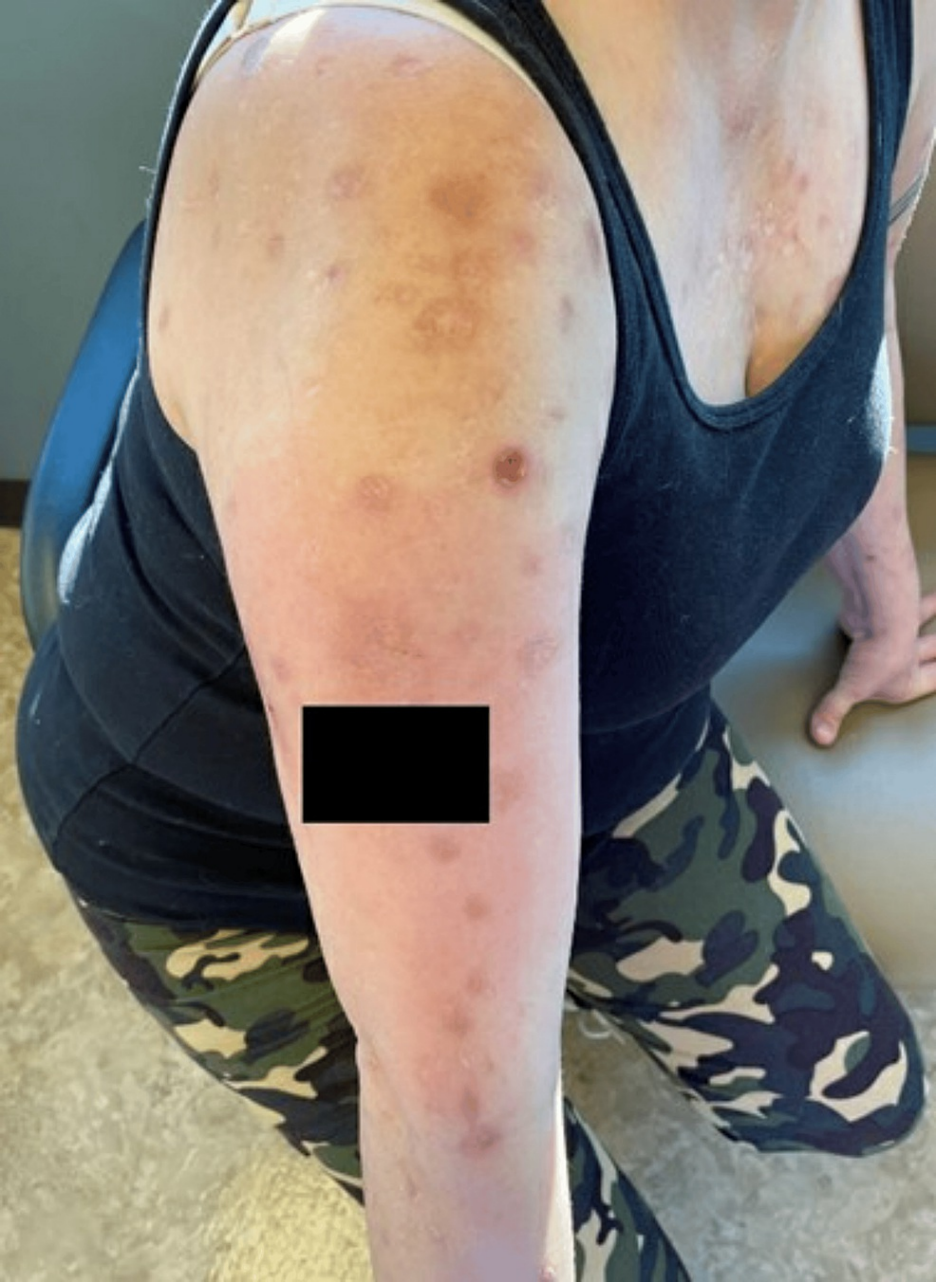
Presentation three months after starting low-dose naltrexone (4.5 mg) There was an improvement in numerous erythematous skin erosions interspersed with areas of scarring, and there were fewer new lesions.

Three months later, the patient returned to the pain clinic for a follow-up. She reported that naltrexone had improved her ED, and she felt less compelled to itch at her skin. On examination, areas of previous erythema and excoriation showed some healing and fewer new lesions (Figure 1). At this visit, she was started on buprenorphine 1 mg BID PO PRN.

**Figure 2:**
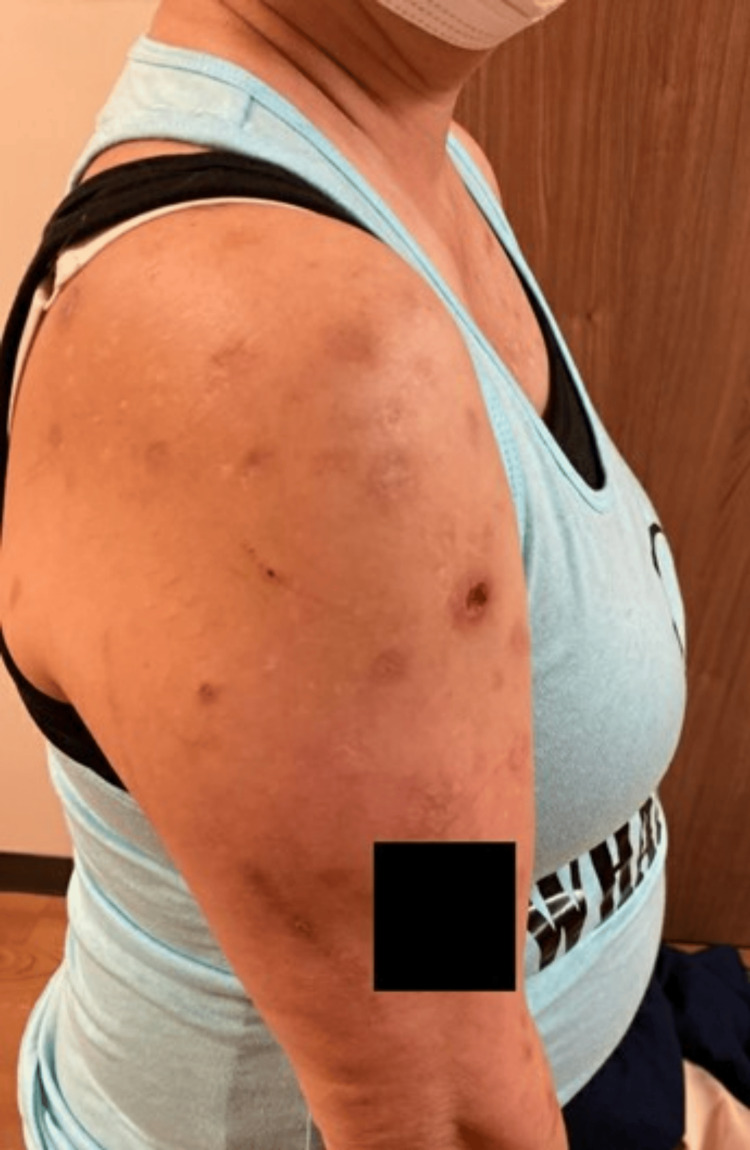
Presentation after one month of interruption of low-dose naltrexone The patient reported a flare in ED, with worsening of skin-picking behavior and development of new erosions.

One month after this visit, the patient was instructed to discontinue naltrexone due to an outpatient procedure scheduled with another medical service. During that time, she left a message stating that her skin-picking disorder worsened once she stopped taking naltrexone (Figure 2). After her procedure was completed, the patient was resumed on naltrexone. When she returned to the pain clinic two months later, she reported subjective improvement in her ED. She sought to continue naltrexone, as she felt that it was helpful in controlling her ED.

## Discussion

This patient experienced a therapeutic benefit from naltrexone for her skin-picking disorder, as demonstrated by the temporal changes in her symptoms. When naltrexone was initiated and then resumed, her ED improved. When naltrexone was interrupted, her ED flared up. 

There are few systematic reviews or randomized control trials that support the use of naltrexone for ED. One systematic review found multiple studies where opioid antagonists were effective in animal models, but opioid antagonists for humans with ED were only effective in case reports [4]. Another systematic review stated that though medications such as naltrexone have been successful in case reports, there are no controlled trials that support their use [5]. Furthermore, other reviews have not found sufficient evidence to support the use of naltrexone in ED. For example, a narrative review of the treatment of trichotillomania, ED, compulsive buying disorder, hypersexual disorder, and binge eating disorder did not support the use of naltrexone [6]. Another systematic review also stated that naltrexone was not an effective treatment option for ED [7]. 

There are case reports that describe naltrexone as a treatment option for ED. In particular, a 50-year-old female with prurigo nodularis presented similarly to our patient. This patient had persistent ED, so she was started on naltrexone 50 mg daily, which demonstrated improvement in her skin-picking behavior [8]. The starting dose was 50 mg, and she was maintained on this dose [8]. Naltrexone has also shown benefits for treating ED in patients with Prader-Willi syndrome (PW). One case described how an approach of fluoxetine and naltrexone improved skin-picking behavior in a patient with PW [9]. Another patient with PW showed near resolution of skin-picking with naltrexone [10]. Naltrexone was started at 25 mg daily for one week and then continued at 50 mg daily. Skin-picking behavior of the patient had nearly resolved within four weeks [10].

Low-dose naltrexone (LDN) has been successfully studied as an immunomodulatory and anti-inflammatory therapy in various dermatologic conditions, including psoriasis, systemic sclerosis, and lichen planopilaris, among others [11]. LDN is an appealing treatment option due to its minimal side effects, low potential for abuse, and cost-effectiveness. There have been reports of self-abusive behavior reversal with HDN, resulting in reduced skin picking and pruritus. This suggests a hypothesis that HDN works by blocking the endogenous reward from the release of opioids, which may occur with excoriation. However, there have been no reports of using LDN in managing ED.

To our knowledge, our case report is the first to describe the efficacy of LDN in managing ED. Our patient was started at a dose of 4.5 mg daily and maintained on this dose. Because she is pleased with her treatment effect, she will be monitored and likely continued on a dose of 4.5 mg.

## Conclusions

To our knowledge, our case is the first description of LDN (4.5 mg) for treating ED. ED has been treated with naltrexone in previous case reports, but these reports frequently utilized doses of 50 mg. One report used a starting dose of 25 mg, which was then later increased to 50 mg. The current literature shows the need for further randomized control trials of ED treatment. Our case provides evidence for the use of naltrexone as an option for trial in the appropriate context.
